# Editorial: The roles of autophagy and cell death in the host immune response in aquatic animals

**DOI:** 10.3389/fimmu.2025.1688035

**Published:** 2025-09-08

**Authors:** Quanyuan Wan, Junji Zhu, Zhendong Qin, Yongyao Yu

**Affiliations:** ^1^ Florida Research and Innovation Center, Cleveland Clinic, Port St. Lucie, FL, United States; ^2^ Guangzhou Key Laboratory of Aquatic Animal Diseases and Waterfowl Breeding, Innovative Institute of Animal Healthy Breeding, College of Animal Sciences and Technology, Zhongkai University of Agriculture and Engineering, Guangzhou, China; ^3^ College of Fisheries, Huazhong Agricultural University, Wuhan, China

**Keywords:** autophagy, cell death, aquatic animal, immune response, pathogens

Autophagy is a cellular mechanism responsible for the degradation of intracellular components, including damaged organelles and proteins, to maintain cellular homeostasis. It plays a pivotal role in the host’s response to infection by eliminating intracellular pathogens and restricting their replication (1). Conversely, certain intracellular pathogens can exploit the host’s autophagy machinery to enhance their replication (1, 2). Furthermore, autophagy can induce cell death, thereby preventing the spread of infection to adjacent cells (3). Emerging evidence indicates that autophagy is a primary mechanism of cell death, known as autophagic cell death, and the interaction between autophagy and cell death in the host’s response to infection is both complex and dynamic. Aquatic animals are exposed to a range of pathogens and environmental stressors that can threaten their health and survival. To counter these threats, aquatic animals possess a sophisticated immune system comprising both innate and adaptive immune responses. Although some research has focused on the induction of autophagy and cell death under pathogen infection in aquatic animals, our understanding of autophagy and cell death in aquatic animal cells is still limited.

In this Research Topic, Cao and Chen elucidate the role of autophagy-mediated neutrophil extracellular trap cell death (NETosis) in the antibacterial defense mechanisms of fish. Their study specifically identifies that infection by *Pseudomonas plecoglossicida* triggers NETosis in the large yellow croaker (*Larimichthys crocea*). Subsequent transcriptome and gene expression analyses reveal an induction of autophagy-related genes and an elevated LC3-II/LC3-I ratio in neutrophils following *P. plecoglossicida* infection. Moreover, the application of the autophagy inhibitor 3-Methyladenine (3-MA) is shown to suppress NETosis, highlighting the essential role of autophagy in the NETosis process induced by *P. plecoglossicida* infection. This research indicates that autophagy-mediated NETosis is a conserved defense strategy against bacterial infections, extending from Teleosts to Mammals. Additionally, xenophagy, a specialized form of selective autophagy in response to pathogen infection, is also examined in aquatic animals. As reviewed by Wang et al., xenophagy serves not only as an innate immune strategy employed by fish hosts to combat aquatic pathogenic bacteria but also as a mechanism exploited by these bacteria for their propagation.

Interestingly, fish also employ microRNA-induced immune responses and potentially autophagy to combat viral infections. Wei et al. have demonstrated that miR-130c-5p in channel catfish (*Ictalurus punctatus*) plays a protective role against Snakehead vesiculovirus (SHVV) infection. In SHVV-infected channel catfish ovary (CCO) cells, miR-130c-5p expression is upregulated. Subsequent studies have revealed that miR-130c-5p not only targets and reduces the expression of the SHVV nucleoprotein (N) gene but also enhances the expression of immune-related genes and activates NF-κB signaling. Notably, among the genes regulated by miR-130c-5p, some are associated with autophagy, suggesting that miR-130c-5p may modulate autophagy to defend against SHVV invasion in catfish.

In addition to initiating an innate immune response, fish also utilize adaptive immunity to eradicate pathogens. IgM is a crucial immunoglobulin in fish. Huang et al. observed that largemouth bass (*Micropterus salmoides*) survived from largemouth bass ranavirus (LMBRaV) infection exhibit a higher survival rate during the second infection compared to wild-type *M. salmoides*, indicating the role of adaptive immune response in viral elimination. Further analysis revealed that LMBRaV-specific IgM possesses viral neutralizing capabilities and facilitates the recovery of *M. salmoides* from LMBRaV infection. Given that autophagy is linked to B cell differentiation and immunoglobulin production (4, 5), it is plausible that autophagy plays a role in the defense of *M. salmoides* against LMBRaV infection. This finding suggests that the relationship between autophagy and immunoglobulin secretion warrants extensive future investigation.

In summary, the studies presented in this Research Topic enhance our understanding of the intricate interactions between autophagy and pathogen infection. These contributions underline the practical significance of autophagy research for controlling pathogenic disease in aquatic animals. Future research should aim to elucidate the detailed mechanisms by which hosts and pathogens manipulate autophagy. Additionally, exploring how autophagy influences the differentiation and maturation of immune cells in aquatic animals would be of interest. Regrettably, research on cell death in aquatic animals is not included in this Research Topic. Given the distinct environmental conditions between aquatic animals and humans, it is worth investigating potential differences in autophagy and cell death between these groups, as well as the evolutionary trajectory of these processes from aquatic animals to humans. We remain eager to learn more about autophagy and cell death in aquatic animals in other Research Topics.
